# *In vitro* cell model to dilucidate the underlying molecular mechanism associated with ophthalmic manifestation of congenital disorders of glycosylation: studying an ALG2-CDG patient

**DOI:** 10.3389/fgene.2025.1678103

**Published:** 2025-10-21

**Authors:** Marisa Angelica Cubilla, Ana Clara Sclausero, Mariano Bisbal, Carla Gabriela Asteggiano

**Affiliations:** ^1^ Núcleo Multidisciplinario de Investigación en Salud Pediátrica de Precisión (NUMISAP)- Hospital de Niños de la Santísima Trinidad, National Council for Scientific and Technical Research (CONICET), Córdoba, Argentina; ^2^ Núcleo Multidisciplinario de Investigación en Salud Pediátrica de Precisión (NUMISAP) Unidad Asociada CONICET - Hospital de Niños de la Santísima Trinidad, Córdoba, Argentina; ^3^ INIMEC National Council for Scientific and Technical Research (CONICET), Instituto de Investigacion Medica Mercedes y Martin Ferreyra, Córdoba, Argentina; ^4^ Universidad Nacional de Cordoba, Córdoba, Argentina; ^5^ Faculty of Health Science, Catholic University of Cordoba, Córdoba, Argentina

**Keywords:** 661W cell model, congenital disorders of glycosylation (CDG), ALG2-CDG, photoreceptor, personalized medicine, N-glycosylation, congenital myasthenic syndrome (CMS), neuromuscular disorder

## Abstract

**Introduction:**

Congenital Disorders of Glycosylation (CDG) are severe disruptions in the synthesis of glycoconjugates, resulting in inherited metabolic conditions. These multisystem diseases, typically inherited in an autosomal recessive manner, have an occurrence rate of approximately 1 in 20,000 to 1 in 50,000 live births. The clinical presentation of CDG is highly varied and complex, with neurological symptoms being predominant, affecting multiple organ systems. The process of glycosylation, a critical post-translational modification, is tightly controlled by proteins encoded by over 250 genes, and mutations in any of these genes are known to cause CDG. The discovery of new associated genes over recent years has accelerated; comprehensively characterizing these, especially rare ones, will aid in identifying novel therapeutic targets, improving prognostic evaluations, and developing effective treatments. In vitro models (such as cell lines or patient-derived “clinical-grade” cells) are essential for advancing CDG research. Notably, 60% of defects affecting N‐ or O-glycosylation impact the eyes, leading to photoreceptor degeneration and cell death. The 661W cell line, derived from immortalized mouse retinal cells and expressing specific ocular markers, serves as a valuable experimental model to study the ocular involvement in CDG.

**Methods:**

In this study, we utilized the 661W cell line to explore the molecular consequences of a homozygous variant in the ALG2 gene (c.752G>T; p.Arg251Leu), which encodes the enzyme α‐1,3‐mannosyltransferase. Following transfection with a plasmid carrying the variants of the gene of interest ALG2 p.Arg251/p.Arg251, we carefully evaluated changes in gene expression using RT‐PCR and Western blotting.

**Results:**

Our results suggest that the 661W cell line may serve as a useful model for examining the potential impact of a specific mutation, supporting a possible link between the mutation’s molecular effects and clinical disease progression.

**Discussion:**

These findings could provide valuable insights to inform the development of targeted therapeutic strategies within the framework of personalized medicine.

## 1 Introduction

*In vitro* models (cell lines or patient-derived cells) have been useful in Congenital Disorders of Glycosylation (CDG) to identify biological processes, investigate and categorize new mutations as disease-causing, and assess protein functionality (Brasil et al., 2018). Glycosylation is a cellular process in which proteins and lipids are modified due to the addition of complex carbohydrates, a post-translational modification common to all eukaryotic cells (Aebi, 2013). Glycans are chains of mono-, poly-, or oligosaccharides that can bind, forming glycoconjugates through the endoplasmic reticulum (ER) and the Golgi apparatus for regulating multiple metabolic processes (Zambon and Muntoni, 2021). In humans, CDG are genetic diseases (1:200/1:50,000) of glycoconjugate metabolism (Cossins et al., 2013; Schachter et al., 2003). This process is highly regulated through encoded proteins for more than 290 genes (Francisco et al., 2019; Cubilla et al., 2023). Defects in N-glycosylation are the most common, and CDG can be divided into 1) protein N-glycosylation defects; 2) protein O-glycosylation defects; 3) glycolipid and GPI anchor synthesis defects; 4) multiple glycosylation pathways and other pathways affected (Brasil et al., 2018; Francisco et al., 2019; Cubilla et al., 2023). More than half of the proteins expressed in all humans are properly glycosylated to fulfill specific functions. The most severe are associated with neurological alterations that present with a deep psychomotor retardation and intellectual disability (Evangelista et al., 2015). A very complex phenotypes that require a multidisciplinary study without which their precise diagnosis is difficult (Jaeken and Péanne, 2017; Al Teneiji et al., 2017) mainly in the early stages of childhood. Still, specific pathologies of a single organ or tissue occur, such as, for example, chondrocytes in osteochondromatosis or certain types of muscular dystrophies (Jaeken and Péanne, 2017; Asteggiano et al., 2018). In the context of this study, we focus on ocular involvement that has been reported in 60% of CDG with defects in N- or O-glycosylation. These patients present degeneration (Gücüm et al., 2021) and death of photoreceptors (Grewal and Hewitt, 2003; Francisco et al., 2018; Tam and Moritz, 2009; Khan et al., 2012; Haro et al., 2018; Uribe et al., 2016). Our group reports a rare type of CDG, due to a homozygous variant in the ALG2 gene, describing the clinical and biochemical phenotype of ALG2-CDG in three Argentinean patients (Thiel et al., 2003; Alcántara-Ortigoza et al., 2024; Papazoglu et al., 2021a). In particular, the ALG2 gene encodes the enzyme α-1,3-mannosyltransferase (EC2.4.1.131) responsible for transferring a Man residue from GDP-Man to the core of forming N-glycans in ER (Man1GlcNAc2-PP-dolichol) (Alcántara-Ortigoza et al., 2024; Papazoglu et al., 2021a). Patients with ALG2-CDG have a multisystem disorder with mental disability, iris coloboma, hepatomegaly, coagulation abnormalities, and defective myelination (Asteggiano et al., 2018), described by Thiel et al. (2003). Mutations in the ALG2 gene were also associated with Congenital Myasthenic Syndrome (Cossins et al., 2013). This affects the protein N-glycosylation pathway in the reticulum and plays an important role in the neuromuscular junction (Engel, 2017). In previous publications, for the first time we characterized the serum glycophenotype of ALG2-CDG patients, analyzing the missense change c.752G>T, p.Arg251Leu, by MALDI MASS (MS) spectra of serum transferrin (serum N-glycans), observing an increase in hyposialylated biantennary and triantennary N-glycans, along with a general increased fucosylation. Immature and processed N-glycans were also detected as defective (Papazoglu et al., 2021a). *In vitro* models (cell lines or patient-derived cells) have been useful in CDG to identify biological processes, investigate underlying molecular mechanisms, categorize new mutations as disease-causing, and assess protein functionality (Sosicka et al., 2022). Primary cultures of fibroblasts from patients constitute relevant experimental biological models in the study of the physiopathogenesis of human pathology, as well as for the testing and validation of potential pharmacological therapeutic strategies, although today they are falling into disuse, given their invasiveness (Valko et al., 2022). Here, we propose the 661W photoreceptor cell line, immortalized cells that express molecular markers, constituting a homogeneous cell line sensitive to light (Uribe et al., 2016; Al-Ubaidi et al., 1992). This line would provide an experimental model for the study of the pathogenesis of ocular involvement due to CDGs. Subcellular protein localization of the expression of genes involved in N- or O-glycosylation has been reported in mammalian retinas, including humans (Thiel et al., 2003). In line with this, here we use the 661W cell line as a model to study the effects of the variant observed in our ALG2-CDG patients in their role in the development of symptoms associated with photoreceptor defects (Gücüm et al., 2021; Papazoglu et al., 2021a; Cubilla et al., 2021; Marquioni-Ramella et al., 2020; Papazoglu et al., 2021b; Morava et al., 2008).

Discovering new genes associated with glycosylation disorders, as well as further studying known genes with low worldwide incidence, will enable the identification of specific therapeutic targets. This progress can facilitate early treatment initiation, improve prognosis, and potentially lead to effective interventions for these established conditions (Chantret et al., 2002; Marklová and Albahri, 2007). In our study, we use dimmuno blotting to evaluate the levels of Alg2 protein and its associated glycans, using lectins WGA and ConA in an ALG2-CDG *in vitro* model (Velazquez-Dodge et al., 2024; Silva, 2019). We observed that mutant ALG2 consistently shows a reduced Alg2 protein expression and lower glycan levels compared to wild-type controls, paralleling findings from previous biochemicalanalyses of patient samples analyzed by IEF and HPLC (Asteggiano et al., 2018; Papazoglu et al., 2021a; Papazoglu et al., 2021b; Bogdańska et al., 2021), despite tissue-specific differences in glycosylation. This suggests that our cell model accurately reflects the glycosylation deficits seen in ALG2-CDG and could serve as a diagnostic tool, capable of detecting subtle decreases in ALG2 glycosylation for future research and clinical applications.

We suggest that this model focuses on how defects in the Alg2 enzyme, which is crucial for N-glycosylation, affect Alg2 protein in the retinal cells, under conditions like retinitis pigmentosa, as observed in patients.

## 2 Materials and methods

### 2.1 In silico analysis

**FIGURE 1:**
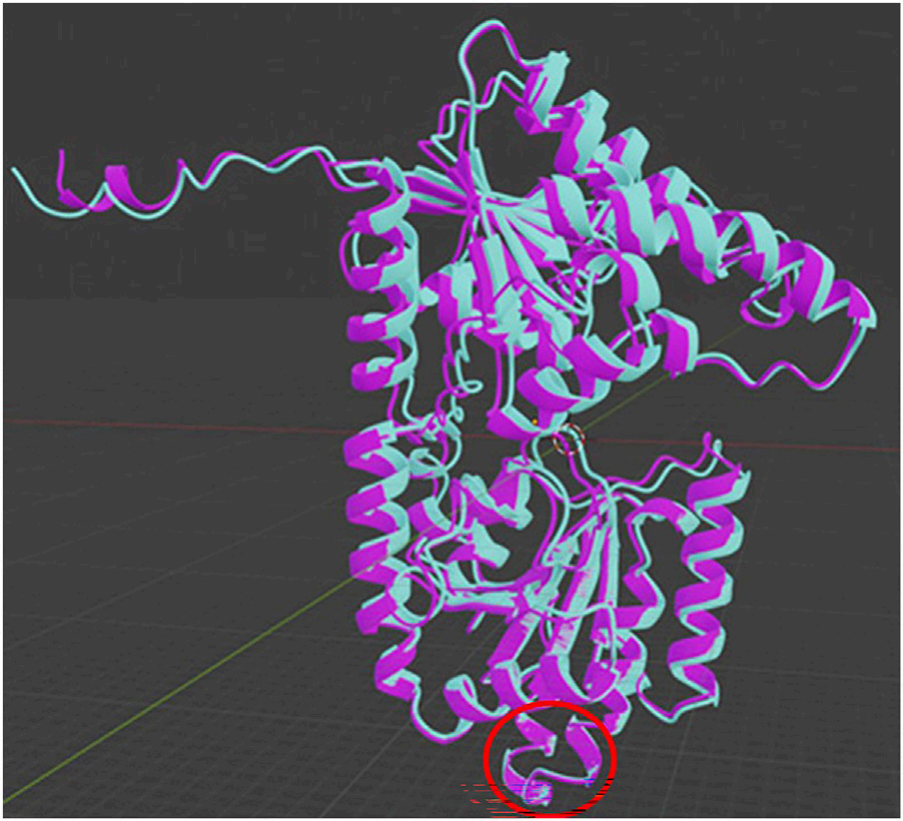
Overlap of the wildtype (light blue) and mutant (purple) forms of the studied ALG2 variant, performed using alpha Foldcolab and Blender. The red ring indicates the location of the analyzed change.

The transcript used to assess the effects of the ALG2 variant (c.752G>T, p.Arg251Leu) is ENST00000476832.2, with the variant ID RS201729325. Using AlphaFold Colab, the protein structure containing the specified variant was modeled, and a Prediction.zip file was generated. This file includes a.pdb file and a predicted_aligned_error.jsonfile. Next, UCSF Chimera, a program for interactive molecular structure visualization and analysis, was used to create a.glb file, which was then imported into Blender. This enabled us to overlay the protein structures on the same plane for a direct comparison and to analyze the structural differences (see Figure 1).

### 2.2 Cell model analysis

To evaluate the functional impact and characterize the cellular changes associated with the ALG2-CDG variant, a cell model was established using HEK293T and 661W cell lines transfected with a homozygous c.752G>T mutation in the ALG2 gene. Wild-type and mutant *ALG2* vectors were kindly provided by Dr. Christian Thiel (Center for Child and Adolescent Medicine, University of Heidelberg) (Thiel et al., 2003).

### 2.3 Transformation of competent bacterium *E. coli* DH5α

#### 2.3.1 cDNA amplification

A piece of the filter paper containing the plasmid carrying the variants of the gene of interest is cut and eluted overnight (ON) using 10 μL of MilliQ water. Competent *E. coli* DH5α bacteria are then prepared for cloning by thermal shock transformation. The plasmid carrying the variants of the gene of interest (1 μL per 100 μL of bacteria) is added to a tube containing bacteria and growth medium (LB: Sodium chloride, yeast extract, and peptone). The mixture is incubated on ice for 20 min, subjected to a 42 °C heat shock for 45 s, and then returned to ice for 5 min.

Afterward, the bacteria are added to LB medium and incubated at 37 °C with shaking for 1 h. The bacteria are then centrifuged at 6,000 rpm for 2 min, and 900 μL of the supernatant is discarded. The remaining bacteria pellet is resuspended in 100 μL of LB medium and plated onto LB agar plates (1.5% agar) containing 100 μg/mL of ampicillin. The plates are incubated at 37 °C overnight. Next, colonies of appropriate size are picked and transferred to a 15 mL tube containing 2–3 mL of LB growth medium with antibiotics. The tubes are incubated in a shaker at 37 °C overnight. The plasmid carrying the variants of the gene of interest from bacterial is then purified using the QIAprep Spin Miniprep Kit, following the manufacturer’s protocol. Finally, the nucleic acid concentration is quantified, and the 260/280 ratio is assessed for purity.

### 2.4 Cell culture

The 661W cell line was generously provided by Prof. Muayyad Al-Ubaidi from the University of Houston. The Human Embryonic Kidney (HEK293T) cells, which express the SV40 large T antigen from the pCMVSPORT6Tag plasmid (Marquioni-Ramella et al., 2020), were kindly donated by Dr. Irazoqui from the National University of Córdoba (UNC), Faculty of Chemical Sciences (FCS). Both cell lines were cultured in DMEM with high glucose (Invitrogen, Carlsbad, CA, United States), supplemented with 100 U/mL penicillin, 100 μg/mL streptomycin, and 10% fetal bovine serum (Life Technologies, Carlsbad, CA, United States). Cultures were maintained at 37 °C in a humidified atmosphere with 5% of CO_2_ and passaged at a 1:5 ratio weekly. The 661W cell medium was additionally supplemented with 2 mM of L-glutamine (Sigma-Aldrich, Argentina) and 1.65 mg of pyruvic acid (Sigma-Aldrich, Argentina). Cells were seeded into six-well plates or 25-cm^2^ flasks at a density of 1 × 10^5^ cells in 3–6 mL of media, and expanded until they reached approximately 50%–90% confluence, depending on the experimental requirements.

### 2.5 Transfection protocol

When the 661W or HEK293T cells reached approximately 70% confluence, they were transfected in duplicate with either ALG2_WT_ and ALG2_mut_ plasmids. Control cells were left untransfected, while a separate group of control cells was treated with the transfection solution without the addition of plasmid DNA.

For transfection, a calcium phosphate precipitate solution was prepared by mixing 2.1 μL of DNA (equivalent to 2.1 μg of cDNA from each plasmid) with 36 μL of a solution containing 274 mM NaCl, 1.8 mM Na_2_HPO_4_, and 50 mM HEPES (pH 7.07). This mixture was then added to the 6-well plates containing the cells. The following day, the medium was replaced with fresh selection media containing DMEM and gentamicin. Cells were harvested 24 h post-transfection for subsequent Western blot analysis and cDNA extraction.

### 2.6 RT-PCR analysis

Total RNA was extracted using TRIzol reagent (Invitrogen). The cultured cells were first washed with phosphate-buffered saline (PBS) and then detached with trypsin. After centrifugation to collect the cells, the pellet was resuspended in an appropriate volume of TRIzol reagent (1 mL per 1–5 × 10^6^ cells) and allowed to lyse at room temperature for 5–10 min. We added 0.4 mL of chloroform per 1 mL of TRIzol. The mixture was vortexed vigorously for 15 s and incubated at 15 °C–30 °C for 2–3 min. After incubation, the sample was centrifuged at 10,000 × g for 10 min at 4 °C. The aqueous phase was carefully transferred to a new tube, and an equal volume of isopropanol (or 0.5 mL per 1 mL of TRIzol) was added. This mixture was then incubated at −20 °C for at least 30 min. Following incubation, the sample was centrifuged at 11,400 × g for 10 min at 4 °C to pellet the RNA. The supernatant was discarded, and the RNA pellet was washed with 70% ethanol (approximately 1 mL per 1 mL of TRIzol) before being centrifuged again at 7,500 × g for 5–10 min at 4 °C. The ethanol was removed, and the RNA pellet was air-dried for 5–10 min before being resuspended in RNase-free water or TE buffer to maintain RNA integrity. The RNA concentration was measured using a spectrophotometer, with an absorbance ratio of A260/280 to assess purity. The RNA was then reverse transcribed into complementary DNA (cDNA) using the Thermo Scientific Revert Aid First Strand cDNA Synthesis Kit (#K1621). An oligo (dT) primer was used to amplify the cDNA of the ALG2 sequence. To evaluate the impact of genetic variants on gene expression of ALG2 the DNA amplification was carried outusing the Polymerase Chain Reaction (PCR) method. The final reaction volume was 25 μL, containing 5 U/μL of Taq DNA polymerase (Invitrogen), 1X reaction buffer, 0.05 mM MgCl_2_, 100 nM of each primer, and 200 μM of dNTPs. The following PCR cycling conditions were used: 2 min at 92 °C, followed by 35 cycles of 1 min at 92 °C, 30 s at 56 °C (the melting temperature, Tm), and 45 s at 72 °C. A final extension step of 10 min at 72 °C was performed, followed by cooling at 26 °C for 15 min. The amplified PCR products were analyzed by electrophoresis on a 2% agarose gel and stained with SYBR Green to visualize the DNA. The primers used are written in Table 1.

**TABLE 1:** Forward (F) and Reverse (R) primers sequence used for the amplification of the ALG2 (exon 2) and GAPDH (exon 7).

Gene	Primers	Melting temperature (Tm)	Size (bp)
ALG2	F: 5′ GGA​TGA​CCT​AGT​CCC​CAA​GG 3′	56 °C	160
	R: 5′ ATA​ACC​ACC​TGC​CAC​GAT​CA 3′		
GAPDH	F: 5′CAC​CAC​CAA​CTG​CTT​AGC​AC 3′	52.5 °C	500
	R: 5′CCC​TGT​TGC​TGT​AGC​CAA​AT 3′		

### 2.7 Western blot

Cell pellets were resuspended in 50 μL of RIPA buffer along with 10 μL of a protease inhibitor cocktail (Roche). The suspension was sonicated for 2 cycles of 10 s each at 50% amplitude, followed by centrifugation for 15 min at 19,000 × g at 4 °C. The protein concentration in the supernatant was determined using the Lowry (Bradford) method (Sigma-Aldrich, St. Louis, MO).

Approximately 20–30 μg of protein was loaded onto 10%–14% polyacrylamide-SDS gels, and electrophoretic separation was conducted for 1 h at 110 V. The proteins were then transferred to a PVDF membrane (GE Healthcare), which had been previously activated with methanol, for 1 h at 240 mA. The membrane was blocked at 4 °C with either 5% milk or 3% BSA and then incubated with the primary monoclonal antibody for 1 h with shaking at room temperature. After 3 washing cycles, the membrane was incubated with a secondary anti-mouse HRP antibody for 2 h at room temperature. Detection was performed using chemiluminescence assays (ECL Plus, GE). Densitometric quantification of the specific bands was performed using ImageJ software. Table 2 lists the antibodies used.

**TABLE 2:** Antibodies.

Antibody	Host	Molecular weight (KDa)	Mark	Dilution
Alg2	Mouse	55	ThermoFisher	1/500–1/100
α-Actina	Goat	43	--	1/1,000
Gliceraldehído-3-fosfato dehidrogenasa (GAPDH)	Mouse	35	(6C5, sc-32233) Biotechnology, Inc. Santa Cruz, CA, EEUU	1/1,000
Peroxidase AffiniPure™ Anti-Human IgG (H + L)	Goat	--	Jackon Inmuno Research	1/5,000

### 2.8 Lectin blots

After transferring the proteins to the membrane, it was washed thoroughly with 1X PBS (pH 7.4). The membrane was then blocked overnight with gentle shaking at 4 °C using 10–20 mL of blocking solution (5% polyvinylpyrrolidone (PVP) in 1X PBS). The following day, the PVP solution was decanted, and the membrane was incubated for 1 h at room temperature (RT) with 0.5 μg/mL of biotinylated lectin, dissolved in 5–10 mL of 1X PBS. The incubation was performed with gentle shaking at RT. After incubation, the membrane was washed with 0.2% TBS-T solution. For band visualization and detection, streptavidin conjugated to IRDye 800 (LI-COR Biosciences) was applied, diluted 1:10,000 in PBS solution, ensuring protection from light. The membrane was then developed using the Odyssey infrared imaging system (LI-COR).

### 2.9 Statistical analysis

Statistics were calculated with GraphPad Prism 6.00 for Windows (GraphPad Software, San Diego, CA; http://www.graphpad.com). The number of samples for each experiment is indicated in the corresponding figure legend. Results were analyzed with one-way ANOVA. Post-hoc Tukey’s test was used for multiple comparisons. Graphs show the significance for comparisons between (controls) and all other conditions, taking Wild Type construct intensity as 100%. Asterisks indicate a statistically significant difference.

## 3 Results

### 3.1 In silico analysis

The missense variant (c.752G>T; p.Arg251Leu), when present in a homozygous state, modifies the DNA sequence, leading to the incorporation of an alternate amino acid. This variant is classified as pathogenic (ClinVar ID 1676197, dbSNP rs201729325) and is of germline origin. Arginine (Arg) is a polar, positively charged amino acid due to the presence of a guanidinium group in its side chain. This group allows arginine to form strong ionic and hydrogen bonds with other molecules, enhancing its solubility in water and its role in protein interactions. In contrast, the variant replaces arginine with leucine (Leu), an apolar and non-polar amino acid characterized by its aliphatic side chain. Leucine is hydrophobic and tends to cluster with other proteins, contributing to the stability of their three-dimensional structures (Figure 1).

### 3.2 Expression after transfection

#### 3.2.1 Gene expression

**FIGURE 2:**
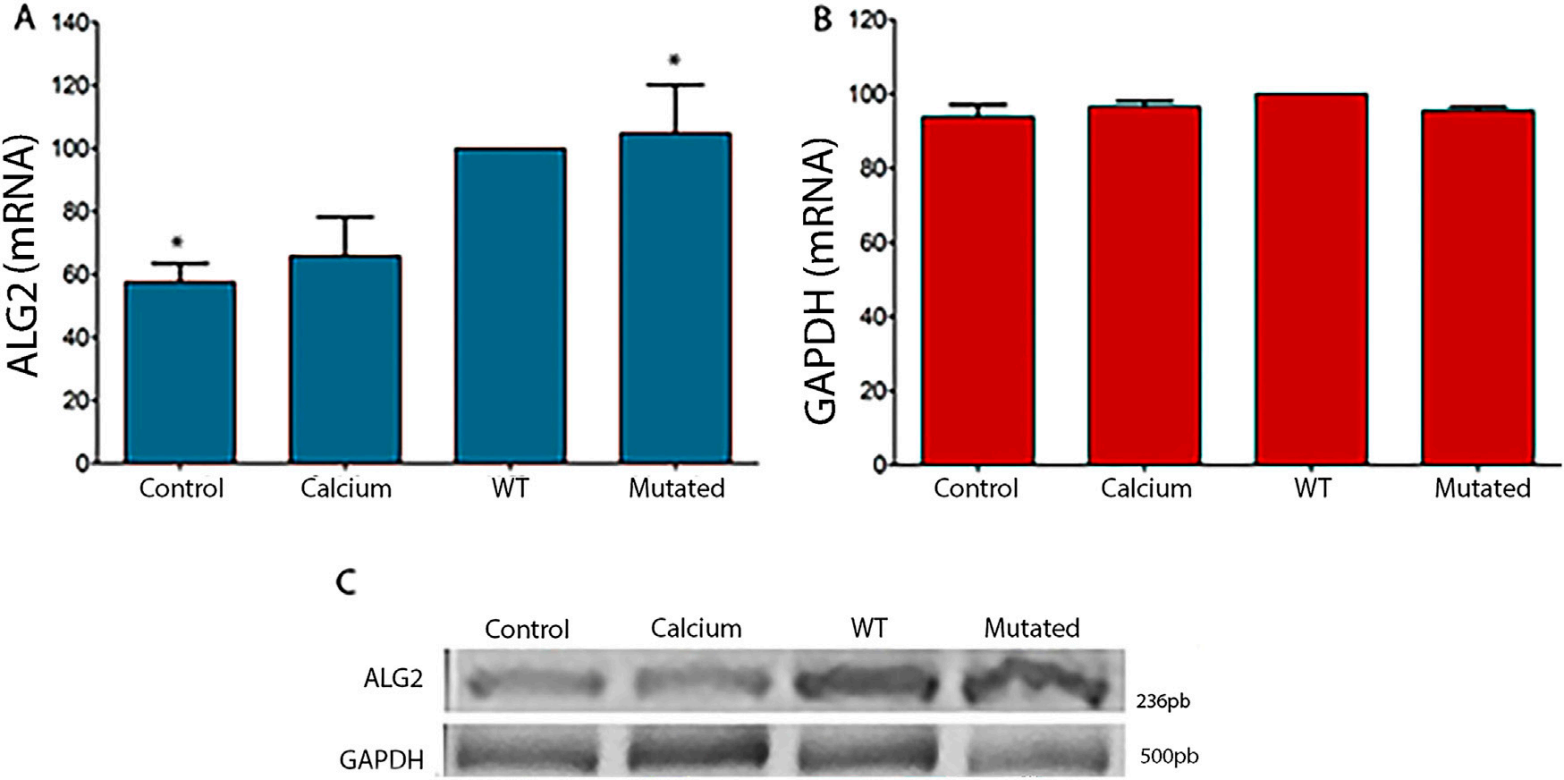
Graphical representation of intensity levels measured with ImageJ software for ALG2 mRNA **(A)** and GAPDH **(B)** in cell cultures of 661W. **(A)** In cultures transfected with the ALG2_wt_ construct (WT), a non-significant increase was observed compared to CONTROL cell cultures and cells treated with calcium phosphate (CALCIUM). A significant increase was observed in cultures transfected with the ALG2_mut_ construct compared to control cultures (P < 0.05). **(B)** No significant differences were observed between the different cell cultures for the GAPDH gene. **(C)** Image of the 2% agarose gel after an electrophoretic run of the cDNAs from each type of 661W cell culture (CONTROL, CALCIUM, Wild Type and Mutated) for ALG2 (top) and GAPDH (bottom). n = 5. Asterisks indicate significant differences.

Cells 661W and HEK293T were transfected with pCI-neo vector containing the Wild Type (ALG2_*wt*_) and the variant sequence of ALG2 (ALG2_*mut.*_). The mRNA levels were analyzed by RT-PCR in untransfected 661W cell cultures (CONTROL), treated with the transfection medium (CALCIUM) but without containing the sequences of interest, and cell cultures containing the sequences of interest (WT and MUT). An increase in levels was observed in cells transfected with the ALG2_wt_ and ALG2_mut_ constructs (significant for this point), a logical result given that they are added to the endogenous levels of the cell line (Figure 2). Expression levels of the housekeeping gene GAPDH were also analyzed. In this case, they remained constant regardless of the cell culture (Figure 2).

**FIGURE 3:**
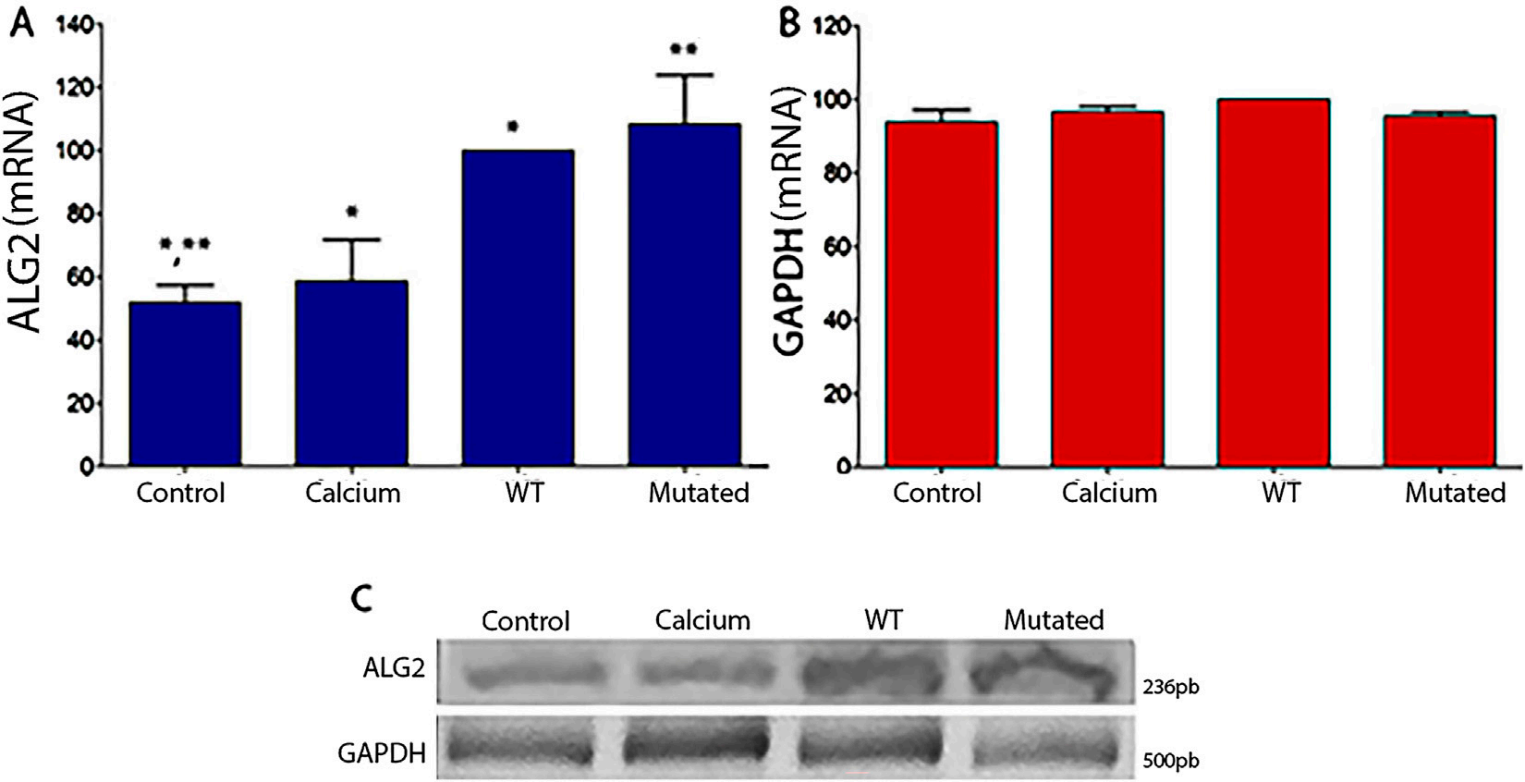
Graphical representation of intensity levels measured with ImageJ software for ALG2 mRNA **(A)** and GAPDH **(B)** in cell cultures of HEK293T. **(A)** In cultures transfected with the ALG2_wt_ construct (WT), a significant increase was observed compared to CONTROL cell cultures and cells treated with calcium phosphate (CALCIUM), (P < 0.05). A significant increase was observed in cultures transfected with the ALG2_mut_ construct (MUT) compared to all cultures (CONTROL, CALCIUM and WT), (P < 0.05). **(B)** No significant differences were observed between the different cell cultures for the GAPDH gene. **(C)** Image of the 2% agarose gel after an electrophoretic run of the cDNAs from each type of HEK293T cell culture (CONTROL, CALCIUM, Wild Type, and Mutated) for ALG2 (top) and GAPDH (bottom).n = 3. A single asterisk (*) denotes a statistically significant difference between Control vs. Calcium and Calcium vs. WT. Double asterisks (**) denote a statistically significant difference between Control vs. Mutated.

For HEK293T cells, the same results were observed. Levels of mRNA were statistically significant in cells with ALG2_wt_ and ALG2_mut_ constructs (Figure 3) in comparison to Controls and Calcium cell cultures. GAPDH levels remained normal.

### 3.3 Protein expression in transfected cells

**FIGURE 4:**
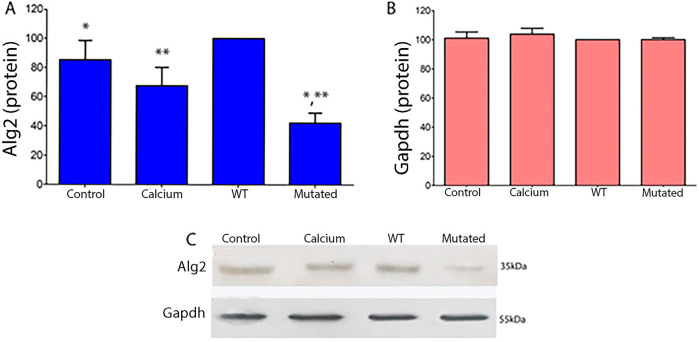
Quantitative protein analysis of Alg2 **(A)** and Gapdh **(B)** Western blots from 661W cell extracts (ANOVA, P < 0.05). **(A)** In WT cell extracts, Alg2 protein levels show a non-significant increase compared to Control and Calcium. However, mutated cell extracts exhibit a significant decrease in Alg2 levels when compared to WT, Control, and Calcium. **(B)** Gapdh levels remain unchanged across all cell extracts. **(C)** The image shows the chemiluminescent plates for each cell culture type (CONTROL, CALCIUM, Wild Type, and Mutated) for Alg2 (top) and Gapdh (bottom), n = 4. Asterisks indicate significant differences: a single asterisk indicates a statistically significant difference between Control and Mutated, while double asterisks denote a significant difference between WT and Mutated.

Protein expression levels in 661W extract cells of Alg2 increased after transfection with the ALG2_wt_ construct, but decreased significantly with the ALG2_mut_ construct. Levels in control cells and those treated with calcium phosphate were at baseline. There were no changes observed for Actin levels (Figure 4).

**FIGURE 5:**
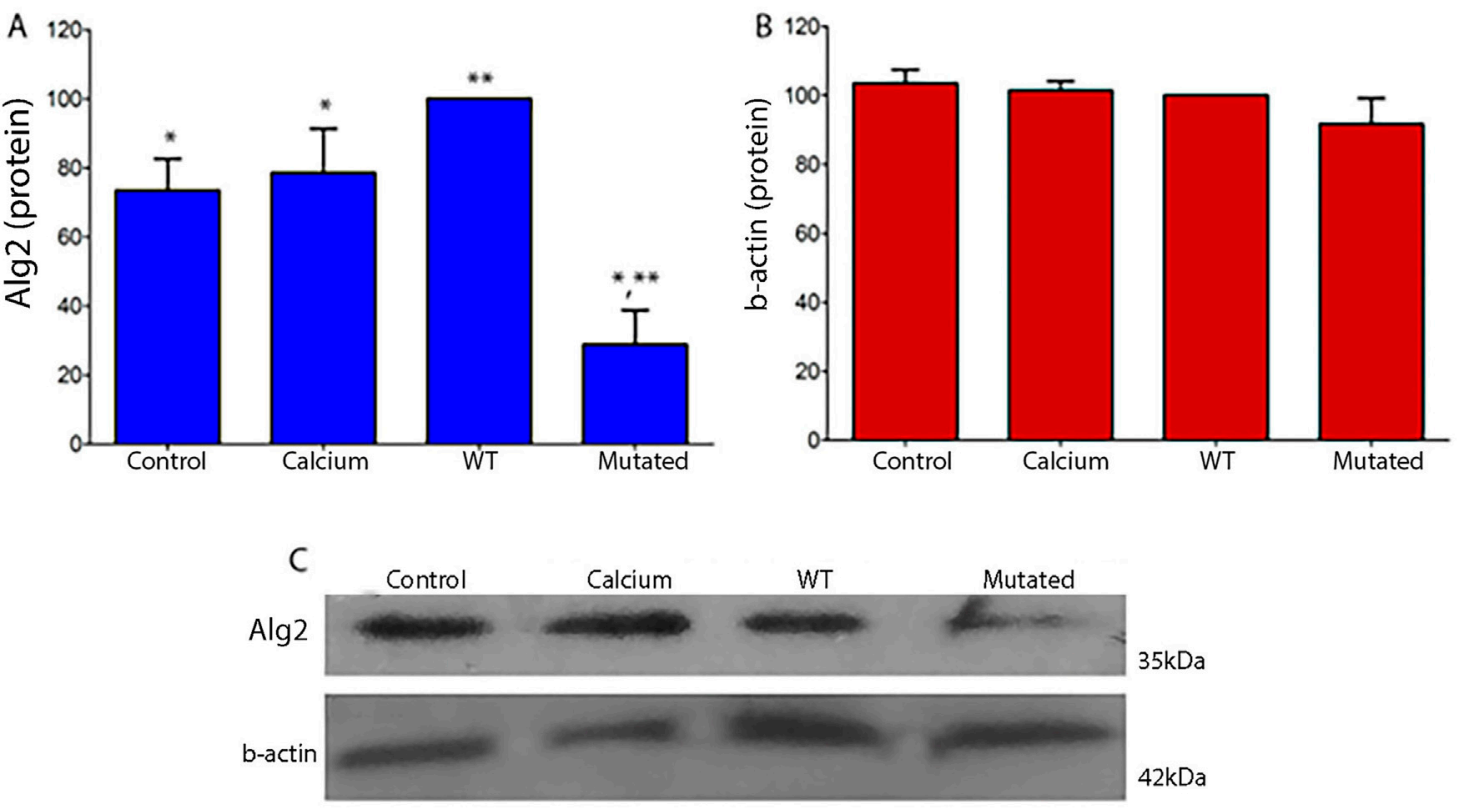
Quantitative protein analysis of Alg2 **(A)** and Actin **(B)** in HEK293T cell extracts (ANOVA, P < 0.05). **(A)** WT extracts show a non-significant increase in Alg2 protein levels compared to Control and Calcium groups. Mutated extracts show a significant decrease in Alg2 levels relative to WT, Control, and Calcium. Actin protein levels remain unchanged in all cell extracts **(B)**. **(C)** Chemiluminescence images show each cell culture type (CONTROL, CALCIUM, Wild Type, Mutated) for Alg2 (top) and Actin (bottom). n = 3. Asterisks indicate significant differences: a single asterisk marks Control vs. Mutated and Calcium vs. Mutated; double asterisks indicate WT vs. Mutated.

In HEK293T cells, the results are similar for protein levels of Alg2. We observed an increase in protein levels in extracts of cells transfected with the WT construct, but a significant decrease in extracts of cells transfected with the Mutated construct with respect to the levels observed in the Control and Calcium cell extracts. There were no differences for Actin levels (Figure 5).

### 3.4 Lectin blots

**FIGURE 6:**
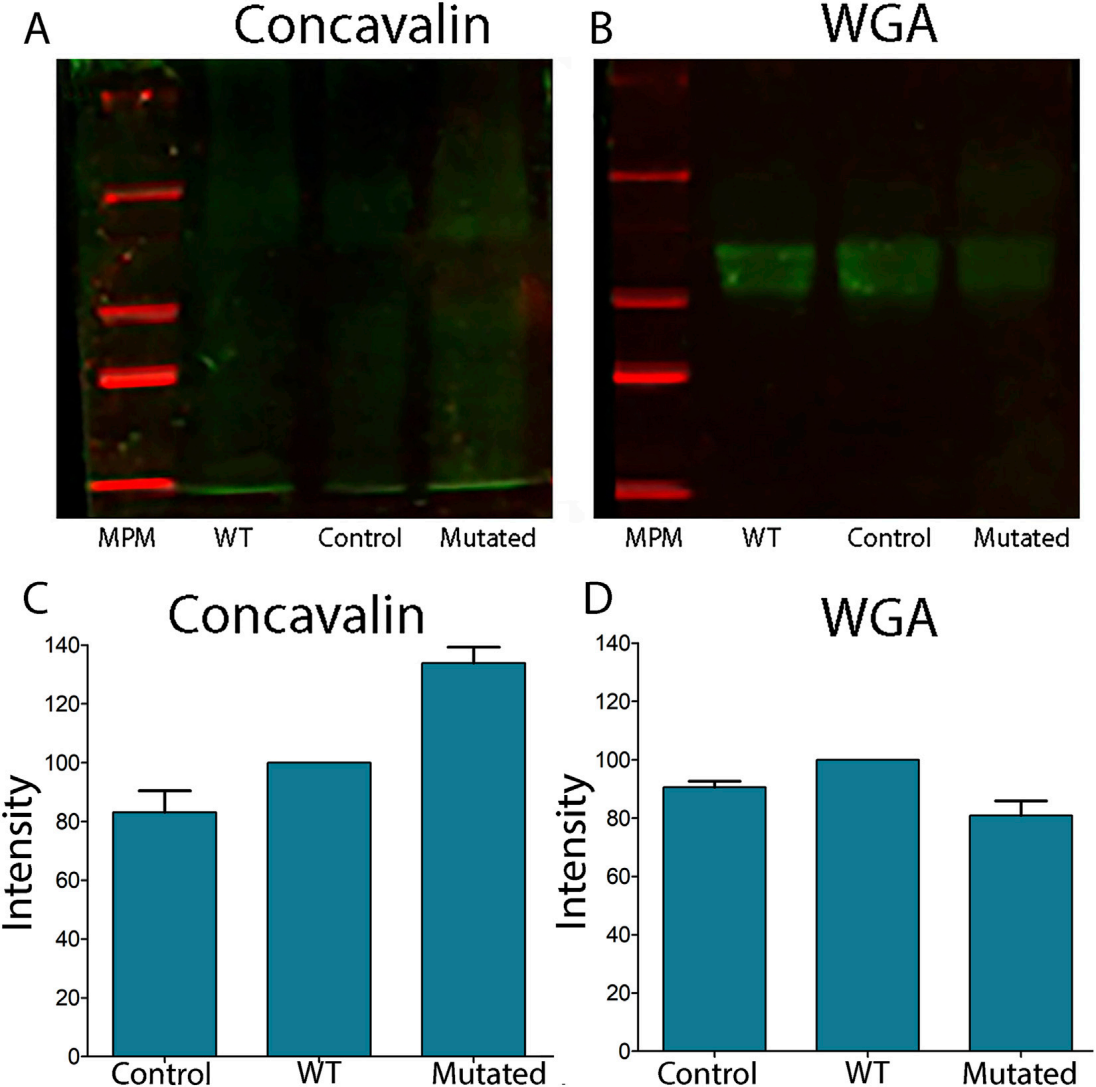
Lectin blots of total extracts of cells 661W transfected with ALG2wt and ALG2mut constructs. **(A)** Concavalin lectin levels showed increased levels in MUT extracts in comparison to WT and CONTROL extracts of cells. **(B)** WGA lectin levels showed a reduction in MUT extracts vs. WT and CONTROL levels. **(C)** Graph of the intensity of the lectin Con A shows an increased level in MUT extracts. **(D)** Graph of the intensity of the WGA lectin shows reduced levels in MUT extracts.n = 3.

Lectins are proteins that can selectively bind to particular glycan structures (Silva, 2019). The lectin Concanavalin A (Con A) binds to mannose and glucose residues, specifically those with unmodified OH groups at positions 3, 4, and 6, and to terminal glucose residues in proteins and peptides. The lectin Wheat Germ Agglutinin (WGA) binds to N-acetyl-D-glucosamine and sialic acid. In order to determine the global levels of protein N-glycosylation, we took advantage of the specific glycan-binding capacity. When we analyzed the lectin content of cell extracts transfected with ALG2-WT and ALG2-Mut constructs by Western blot, we observed increased mannose levels detected in ConA (133% in MUT vs. 100% in WT) and reduced N-acetylglucosamine levels detected in WGA (100% in WT vs. 80% in MUT) (Figure 6). However, apart from these percentage changes, the differences were not statistically significant.

## 4 Discussion

Morava et al. (2008) described the ophthalmic involvement in N-glycosylation disorders, and Francisco et al. (2018) reviewed the ophthalmic aspects of CDG with pure O-glycosylation defects (Francisco et al., 2018; Morava et al., 2008). Ophthalmological anomalies are common in CDGs. They occur in both anterior and posterior segments of the eye, but the occurrence becomes apparent with age. Optic atrophy, cataract, retinitis pigmentosa, strabismus, nystagmus, iris coloboma, and chorioretinal coloboma are indeed frequent (Morava et al., 2008). A routine screening and follow-up for all children diagnosed with CDG could be very important for an adequate treatment, and an early correction with surgery could be successfully achieved, ensuring a better visual development and improving the quality of life (Morava et al., 2008). While various models are employed to investigate the molecular mechanisms underlying cellular alterations induced by different CDG, the subcellular aspects of ophthalmological involvement remain poorly characterized. Esfandiari et al. (2019), investigated the pathogenic ALG12 variant p.C188Y and reported a phenotype characterized by acquired early-onset cataracts, deep-set eyes, and retinitis pigmentosa (Esfandiari et al., 2019). In general, there is little description at the ophthalmological level for CDG Type I.Patients with PMM2-CDG variants have significant photoreceptor dysfunction at the time of ERG (electroretinograms) recording, and the amplitudes of the waves are markedly attenuated (Thompson et al., 2013). Midena and Pilotto (2022), reported multiple retinal astrocytic hamartomas as a retinal finding in an adolescent affected by a congenital disorder of CDG-Ia (Midena and Pilotto, 2022). There are genetic diseases related to the synthesis of Dolichol that are classified as CDG-I (the class of CDG that involves defective assembly of glycans and/or their transfer in the endoplasmic reticulum [ER]). In these diseases, defects in the photoreceptors are also observed, as in retinitis pigmentosa (Ramachandra Rao et al., 2020; Morava et al., 2010).

Gücüm et al. (2021), established a medaka model to investigate the function and regulatory interaction of the complex glycosylation network. They observed that, although rod photoreceptors are originally developed in ALG2 mutants, they are not maintained overtime. These findings suggest thatthe downregulation of certain proteins may represent direct Alg2 targets whose glycosylation is essential for rod photoreceptor survival (Gücüm et al., 2021; Srinivasan et al., 2015).

Very few studies have been conducted on CDG-associated genes in 661W cells. It is crucial to gain a better understanding of the expression and distribution of these proteins in this cell line and in the retina to clarify their role in the development of ocular symptoms linked to these pathologies.

In this paper, the 661W cell line, a model derived from murine cone photoreceptors,is presented as a promising model for ophthalmological studies. The 661W cell line expresses the simian virus 40 (SV40) large T-antigen under the control of the human interphotoreceptor retinoid-binding protein (IRBP) promoter, which is specifically active in photoreceptor cells and encodes a retinol-binding protein (Valko et al., 2022). 661W cells are known to express several markers specific to cone photoreceptors, including cone opsins, cone arrestin, and transducin (Tan et al., 2004). They also demonstrate cell death pathways in response to photooxidative stress that are similar to those observed in native retinal photoreceptors (Kanan et al., 2007). Morphologically, these cells exhibit neuron-like, spindle-shaped processes. Due to these properties, 661W cells serve as a reliable *in vitro* model for studying the biology of cone photoreceptor cells and retinal degenerative diseases. Furthermore, their progenitor-like phenotype makes them particularly valuable for evaluating neuroprotective strategies aimed at promoting photoreceptor survival in conditions such as cone dystrophies, age-related macular degeneration (AMD), and retinitis pigmentosa (RP) (Sheedlo et al., 2007). There are few studies that address this cell type in specific, especially the variant studied in this research. Knowing its existence and function could perhaps help to identify markers prior to the onset of disorders associated with this variant. This allows researchers to analyze enzyme function, assess glycosylation patterns, and test potential therapies.

In this research, we specifically studied the human ALG2 gene that encodes an α 1,3mannosyltransferase that catalyzes the first steps in the synthesis of N-glycans in the endoplasmic reticulum (Haro et al., 2018; Uribe et al., 2016). The specific interest in ALG2-CDG is due to our previously reportedresearch on three patients carrying a c.752G>T, p.Arg251Leu ALG2 missense variant in a homozygous state (Alcántara-Ortigoza et al., 2024). Our previousstudies showed an increase in serum hyposialylated biantennary and triantennary N-glycans of all patients, along with an overall increase in fucosylation, through a MALDI MASS (MS) spectra analysis of serum transferrin N-glycans and total serum N-glycans. We observed that immature and defectively processed N-glycans, such as high-mannose, hybrid, and hypogalactosylated species, were also detected (Papazoglu et al., 2021a).

However, the challenge in our research of 2021 (Papazoglu et al., 2021a) was to characterize the specific cellular changes caused by the pathogenic variant in ALG2-CDG patients. We found that, similar to what was observed in serum glycoproteins, this variant causes a systemic defect that produces affected N-glycans at the cellular level when compared to control cell lines (Cubilla et al., 2023; Uribe et al., 2016). We can observe this variant in amino acid 251 of the protein Alg2. This position does not correspond to areas typically predicted to be transmembrane, which are usually located closer to the N-terminus and C-terminus. The variant resides in a region oriented towards the lumen or cytosol. Therefore, this variant falls within the ALG2 functional domain, which may affect mannosyltransferase activity. Furthermore, the change in the charge on the amino acid (from polar to apolar) may alter its local structure, stability, or interaction with putative substrates/lipids. So, although the variation is not located within a transmembrane, we could predict thatit is placed within a critical domain essential to its normal biological function. Programs such as Polyphen, SIFT (Sorting Intolerant from Tolerant), and MutPred classify this variant as possibly harmful, deleterious, and potentially affecting the local function by disrupting protein-protein interaction sites or structural stability.

The lectin studies showed an ALG2 deficiency that could disrupt the formation of more complex glycans, interfering with the correct functioning of the glycoproteins involved (Haro et al., 2018; Uribe et al., 2016; Papazoglu et al., 2021a). Studies of total serum N-glycans and serum Tf-specific studies carried out in our laboratory show altered N-glycan biosynthesis in ALG2-CDG patients (Alcántara-Ortigoza et al., 2024). The presence of monosialylated glycans and the increase in fucosylated forms and truncated isoforms suggest an alteration in the biosynthetic pathway of oligosaccharides linked to dolichol, prior to the union of the glycan group or core to the protein (Morava et al., 2009). In this paper, we showed alterations in the lectin profile for Con A and WGA in cell expressing mutant ALG2 Plasmid (Gücüm et al., 2021). The results obtained indirectly support the idea that the gene variants found in these patients lead to alterations in the glycosylation pathway, causing incomplete glycan chains on cell-surface glycoproteins (Velazquez-Dodge et al., 2024; Srinivasan et al., 2015).

Moreover, the results indicate that the ALG2 variant overexpressed in HEK-293 and 661W cell lines lead to a marked reduction in protein levels, suggesting a potential toxic gain-of-function capable of overriding endogenous ALG2. Unlike most ALG2 mutations, which typically cause loss of function, this variant appears to operate via a distinct pathogenic mechanism (Thiel et al., 2003; Papazoglu et al., 2021a).

The presence of this analyzed variant may trigger Alg2 degradation by disrupting proteins involved in its proper folding, Golgi trafficking, or degradation signaling (Gücüm et al., 2021; Meunier et al., 2002). Disruptions in the glycosylation machinery can lead to endoplasmic reticulum (ER) stress (Lee, 2005), which, resulting from defective N-glycosylation, is known to trigger apoptosis (Szegezdi et al., 2006). To validate these proposed mechanisms, it is necessary to relay on functional assays, assessment of the protein’s intracellular localization, and more advanced experimental models. These approaches will help elucidate the specific process underlying the reduction in Alg2 levels, even in the presence of endogenous Alg2 WT.

Interestingly, ALG2 has been reported to encode a bifunctional glycosyltransferase enzyme activity and is responsible for the transfer of the α1,3- and α1,6-mannose linked residue from GDP-mannose to Man1GlcNAc2-PP-Dol, forming the Man3GlcNAc2-PP-Dol intermediate on the cytosolic side of the ER. This is essential for the biosynthesis of N-linked glycoproteins (Reily et al., 2019). The specificity of glycan synthesis might well be subject to spatial and temporal regulation within the Golgi during various developmental and disease states (Al Teneiji et al., 2017; Alcántara-Ortigoza et al., 2024). Therefore, a slight Golgi functional abnormality, already observed in other Type I CDGs such as ALG12-CDG, could also be suggested (Sturiale et al., 2011). In particular, it can be hypothesized that, as demonstrated for 1,6-mannosyltransferase (ALG12) deficiency (Quintana et al., 2009), the observed increased amounts of high-mannose and hybrid species may well be attributed to the alpha-1,3/1,6-mannosyltransferase (ALG2) molecular defect, since disruption of both enzymes, acting in the first step of the ER N-glycosylation pathway, showed an accumulation of such abnormal protein-linked structures (O’Reilly et al., 2006). This allows as to hypothesize a synergistic effect between both enzymes.

Finally, we could demonstrate the usefulness of this cellular model (661W) for assessing the effects of gene variants associated with CDG. Given that ALG2 is one of the fourteen genes implicated in Congenital Myasthenic Syndrome, a deeper understanding of tissue-specific pathophysiology may help link clinical manifestations to ocular involvement in CDG patients. Expanding the knowledge of the ocular etiopathogenesis of CDG will contribute to a better understanding of the disease’s natural history, help informpotential treatment and prognosis,and support timely genetic counseling, including opportunities for early intervention (Brasil et al., 2018; Stavenhagen et al., 2022).

## Data Availability

The original contributions presented in the study are included in the article/supplementary material, further inquiries can be directed to the corresponding author.
